# The Prophylactic Use of Lipid Emulsion Therapy in the Excision of Invasive Malignant Melanoma under Local Anaesthetic in a Morbidly Obese Patient

**DOI:** 10.1155/2013/765279

**Published:** 2013-01-16

**Authors:** K. S. Sharma, P. Lim, T. M. Brotherston, P. Smith

**Affiliations:** ^1^Royal Hallamshire Hospital, Sheffield Teaching Hospitals, Glossop Road, Sheffield S10 2FJ, UK; ^2^Department of Plastic and Reconstructive Surgery, Sheffield Teaching Hospitals, 4 Claremont Place, Glossop Road, Sheffield S102 FJ, UK; ^3^Nottingham University Hospitals, Nottingham NG5 1PB, UK

## Abstract

We present the first reported case of the prophylactic use of lipid emulsion therapy in the removal of an extensive, circumferential malignant melanoma in a morbidly obese patient, under local anaesthetic. The advantages of this technique allowed the patient to avoid intraoperative invasive monitoring and postoperative critical care admission and assisted during the operation by rotating her leg when needed. This is a useful technique that can be employed in urgent cases where there is a need to excise extensive skin malignancies in patients who are unsuitable for general or regional anaesthesia.

## 1. Introduction

The increasing incidence of obesity accompanied with its associated health risks means that more patients who are unfit for general or regional anaesthesia will present for surgery. Between 1993 and 2010 the prevalence of morbid obesity increased from 1.5% to 3.8% in women [1]. Their perioperative care presents a challenge to the anaesthetic and surgical teams. Increased concentrations of triglycerides, lipoproteins, cholesterol, and free fatty acids in these patients alters the protein binding of anaesthetic agents increasing their plasma concentrations [2]. Furthermore obesity is often associated with impaired hepatic function, which modifies drug metabolism and clearance [3]. These all increase the risk of local anaesthetic toxicity.

## 2. Case Report

A 65-year-old morbidly obese patient (BMI 64) presented with a thirty-year history of circumferential venous ulcers of her left lower leg, treated with compression bandages. A curetting biopsy revealed a malignant melanoma of uncertain Breslow thickness and a mitotic rate of three per square mm. After discussion among the specialist skin cancer multidisciplinary team, she was referred to our Plastic Surgery Unit for advice regarding surgical management.

**Figure 1:**
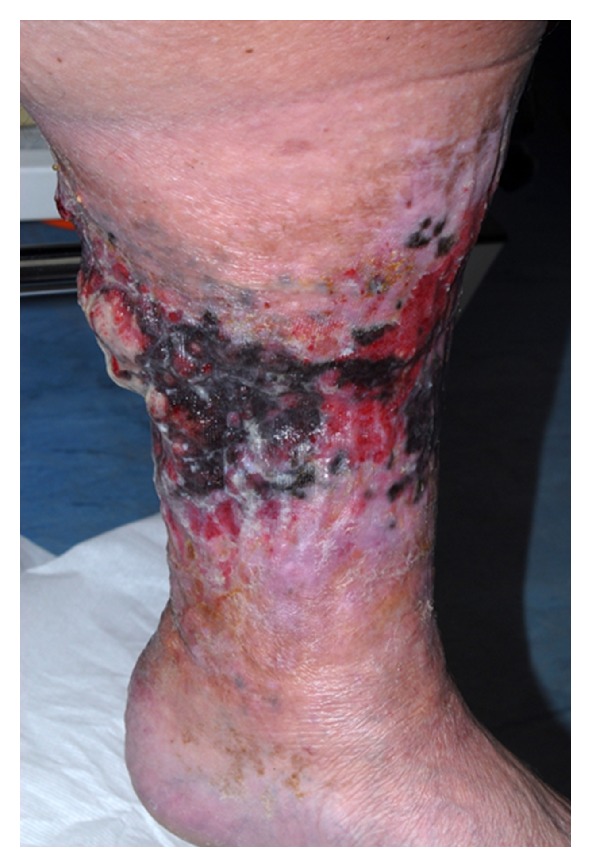
Circumferential malignant melanoma to the left lower leg demonstrating its extent.

Examination revealed a circumferential ulcer extending from above the ankle to below the knee. It was eroded anteriorly with surrounding areas of dark discoloration and satellite nodules (Figure 1). She had no palpable lymphadenopathy. CT scan of the head, abdomen and pelvis revealed several small bilateral external iliac lymph nodes of uncertain significance.

The possible options of excision and reconstruction versus amputation were discussed with the patient. She opted for excision and reconstruction with a split thickness skin graft. Past medical history included atrial fibrillation, ischaemic heart disease, and pulmonary embolus. A preoperative echocardiogram revealed a pulmonary artery pressure of 63 mmHg, which excluded general or regional anaesthesia [4]. After discussion with the patient it was decided to attempt the operation under local anaesthesia in combination with a prophylactic infusion of lipid emulsion therapy (Intralipid Fresenius Kabi, Uppsala, Sweden) and sedation.

Under full monitoring 100 mls of 20% intralipid was administered intravenously. Light sedation was commenced with propofol 1% at 5 mls/hour and remifentanil at 125 mcg/hour. A ring infiltration proximal to the lesion was performed with a mixture of 20 mls of 0.5% lidocaine and 1 in 200,000 adrenaline and 80 mls of 0.25% levobupivacaine and 1 in 200,000 adrenaline all in combination with 1,500 international units of hyaluronidase (Hyalase, Wockhardt, Wrexham, UK). 15 mls of 0.25% and 15 mls of 0.125% levobupivacaine were additionally injected during the procedure. After a further 100 mls of 20% Intralipid four hours after the initial injection, the donor sites were infiltrated with 80 mmls of 0.125% levobupivacaine and 1 in 200,000 adrenaline in combination with 1500 international units of Hyalase. The patient was continuously monitored for early symptoms of local anaesthetic toxicity and assisted surgical access by internally or externally rotating her leg as appropriate. Postoperatively she had a 45-minute stay in recovery before discharge back to the ward and required no opioids.

Histology revealed an invasive nodular malignant melanoma; clinical stage 3B with a Breslow thickness of 21 mm and Clark's level four. The tumor was completely excised with 3 mm clear margins. At two-month review postoperatively the grafts and donor sites were well healed, and she was discharged into the care of the oncologists for further treatment.

## 3. Discussion

Local anaesthesia is safely used for many plastic surgery procedures limited by the dose that can be administered before plasma concentration causes systemic toxicity. As long as inadvertent intravascular injection is avoided, the plasma concentration reflects the rate of absorption less than the metabolism. Local anaesthetic systemic toxicity (LAST) occurs in a well-defined clinical sequence and includes tinnitus, lightheadedness, and circumoral tingling. At higher plasma levels unconsciousness, respiratory arrest and cardiovascular depression can occur [5].

The absorption of local anaesthetic varies by injection site, being highest after intercostal blocks followed by epidural/caudal, brachial plexus, femoral/sciatic block and lowest when injected subcutaneously. This reflects the vascular supply of each tissue. Despite different tissue absorption rates, the maximum dose recommendations are presented as exact milligram per kilogram (mg/kg) doses.

20% of intralipid is an emulsion consisting of 200 g of purified soybean oil, 12 g purified egg phospholipids and 22 g anhydrous glycerol. It has been in medical use since 1962 as a source of omega-3 and -6 fatty acids in parenteral nutrition [6]. Its use in the treatment of LAST was a chance discovery by Weinberg in 1998 and is now part of the treatment pathway for the management of local anaesthetic-induced toxicity in the United Kingdom [7, 8]. It is believed that intralipid forms a “lipid sink,” that is, an expanded intravascular lipid phase that acts to absorb the circulating lipophilic drug which reduces the amount of unbound free drug to bind to the myocardium [6]. In addition to its use in LAST, lipid emulsion therapy has been successfully used in the treatment of toxicity from other lipophilic drugs like chlorpromazine, beta-blockers, and calcium channel antagonists [9].

The maximum recommended dose for levobupivacaine is 2 or 3 mg/kg or 150 mg. We used 350 mg over a four-hour period, which was almost two and a half times the recommended maximum dose. Although this is a large quantity, it is less than what is reported to be safe in tumescent anaesthesia where up to twenty times the maximum local anaesthetic dosage is used [10]. The incremental nature of the injections spreads the absorption, and unlike liposuction significant quantities of the local anaesthetic were excised within the specimen.

Did we need the intralipid? We cannot say for sure, however, given it has minimal side effects that it seemed prudent. There is also a possibility that the morbidly obese might behave as a tumescent patient due to increased subcutaneous fat and odema.

The use of Hyalase is controversial in cases of malignancy; however, literature review revealed no evidence of definitive spread of malignant cells. Additionally the ring block was administered remotely from the tumor and not into the lesion itself.

This is the first reported use of intralipid prophylactically in this subgroup of patients in world literature. Anaesthesia of the required area, tumor clearance, and healed wounds were achieved without any reported significant anaesthetic side effects. This could be a useful addition to the armamentarium in plastic surgery. In the unfit patient with multiple comorbidities the described technique can be used to achieve anaesthesia of the required area, avoiding the risks of invasive anaesthetic procedures.
